# Effects of Exercise on Post-Stroke Depression: A Systematic Review and Meta-Analysis of Randomized Controlled Trials

**DOI:** 10.3390/life15020285

**Published:** 2025-02-12

**Authors:** Yifan Zhang, Gen Li, Wenda Zheng, Ze Xu, Yuanyuan Lv, Xiaojie Liu, Laikang Yu

**Affiliations:** 1 Beijing Key Laboratory of Sports Performance and Skill Assessment, Beijing Sport University, Beijing 100084, China; 18738314013@163.com (Y.Z.); sunflowerlyy@bsu.edu.cn (Y.L.); 2 Department of Strength and Conditioning Assessment and Monitoring, Beijing Sport University, Beijing 100084, China; zwd669966@163.com (W.Z.); xz2000413@163.com (Z.X.); 3 School of Physical Education & Sports Science, South China Normal University, Guangzhou 510631, China; li287270242@163.com; 4 China Institute of Sport and Health Science, Beijing Sport University, Beijing 100084, China; 5 Department of Pharmacology and Toxicology, Medical College of Wisconsin, Milwaukee, WI 53226, USA

**Keywords:** exercise, multicomponent training, stroke, post-stroke depression

## Abstract

Numerous studies have investigated the effects of exercise on post-stroke depression (PSD), yet the findings remain inconclusive. This study aims to evaluate the impact of exercise on depressive symptoms in stroke patients and to identify the most effective exercise protocols for this population. A systematic review of the Embase, PubMed, Cochrane Library, Web of Science, and Scopus databases was conducted, with a search cutoff date of 13 September 2024. Quantitative synthesis was employed to assess the intervention effects, with effect sizes expressed as standardized mean differences (SMDs) and 95% confidence intervals to evaluate the efficacy of exercise in alleviating PSD. A total of 24 studies met the inclusion criteria. The results indicated that exercise significantly alleviated depressive symptoms in stroke patients (SMD = −0.18; *p* = 0.007). Specifically, multicomponent training emerged as the most effective intervention for reducing depression (SMD = −0.24; *p* = 0.008). Additionally, exercise programs with a duration of ≥12 weeks (SMD, −0.17; *p* = 0.04), ≥3 sessions per week (SMD, −0.20; *p* = 0.02), <60 min per session (SMD, −0.19; *p* = 0.05), and <180 min per week (SMD, −0.27; *p* = 0.02) were found to be the most effective in alleviating PSD. Exercise represents an effective strategy for managing PSD, with multicomponent training potentially serving as the optimal intervention. These findings provide evidence for clinicians, recommending that stroke patients engage in exercise at least three times weekly, with individual sessions not exceeding 60 min. By increasing the frequency of exercise, the cumulative weekly time should ideally remain below 180 min for optimal outcomes.

## 1. Introduction

Stroke stands as the second leading cause of mortality and the third leading cause of disability globally, with its burden exhibiting notable socioeconomic disparities [1]. In high-income countries, approximately 20% of individuals will experience a stroke at least once in their lifetime, whereas in low-income countries, this proportion may climb to nearly 50% [2,3]. In 2019, the worldwide total of stroke patients surpassed 100 million, inclusive of 12.2 million new cases [4]. Epidemiological models predict that the annual number of new stroke cases will continue to escalate at a rate of roughly 20% per year, potentially reaching 15 million new cases by 2024 [3]. Based on their pathological mechanisms, strokes can be categorized into ischemic and hemorrhagic strokes, with causes encompassing large-artery disease, small-vessel disease, and cardiac embolism. Identified risk factors include environmental factors like air pollution, lifestyle factors such as smoking, alcohol misuse, poor dietary habits, and physical inactivity, as well as conditions such as hypertension, diabetes, hypercholesterolemia, cardiovascular diseases, and obesity [5,6].

Stroke patients often exhibit a spectrum of functional impairments ranging from motor disorders like hemiplegia and muscle weakness and cognitive impairments such as speech difficulties and memory loss to emotional disturbances, including anxiety and depression [7]. Among these, post-stroke depression (PSD) is the most prevalent neuropsychiatric disorder, affecting 20% to 60% of stroke patients, and can it manifest during either the acute or recovery phase [8]. The pathogenesis of PSD is primarily associated with neuroinflammation and neurotransmitter imbalance [9]. Additionally, functional impairments stemming from stroke can adversely affect a patient’s self-esteem, social engagement, and capacity to perform activities of daily living (ADL), thereby exacerbating depressive symptoms. Research indicates that PSD not only impedes the recovery of motor and cognitive functions but also elevates both mortality and recurrence risks [10,11,12]. Stroke patients with depressive symptoms face a threefold higher risk of death over the subsequent ten years compared with those without depressive symptoms [13].

Current treatment strategies for PSD encompass a variety of pharmacological interventions, including antidepressants, anti-inflammatory drugs, and neuroprotective agents [14]. Additionally, psychosocial therapies, such as cognitive behavioral therapy and interpersonal therapy, are utilized to provide comprehensive care [15,16]. However, research has demonstrated that antidepressants exhibit moderate effectiveness for mild to moderate depression and may include side effects that impact the central nervous and gastrointestinal systems [17]. Similarly, the therapeutic benefits of psychotherapy tend to be modest [18]. In this context, exercise interventions have garnered growing interest as a potential alternative treatment. Previous studies suggest that exercise may alleviate depressive symptoms by fostering motor-function recovery, enhancing cortical brain activity, and improving neural responsiveness [19,20,21].

Existing research indicates that exercise interventions are efficacious in treating depression in non-stroke patients, with a standardized mean difference (SMD) of −0.82, suggesting a moderate to large effect size [22]. In addition, in patients with depression, exercise interventions exhibit a medium to high effect in alleviating depressive symptoms [20]. In stroke populations, various studies have explored the effects of different exercise protocols. For instance, Maček et al. [23] demonstrated that a 3-week multimodal exercise training program significantly improved PSD symptoms, while Lapointe et al. [24] confirmed the positive effects of 18 weeks of high-intensity interval training (HIIT). However, contrasting results have also been reported. Jun et al. [25] found that a music-based exercise intervention improved PSD symptoms, whereas studies by Vloothuis et al. [26] and Ihle-Hansen et al. [27] observed no significant improvement in PSD symptoms following exercise interventions.

Although a meta-analysis published in 2014 investigated the effects of exercise interventions on PSD [28], it suffered from a small sample size and potential selection bias. Furthermore, a meta-analysis examining home-based training for PSD included joint flexibility training, which had a limited impact on physical functioning, thereby suggesting some reservations about justifying its inclusion in the exercise intervention group [29]. Moreover, another meta-analysis exploring the impact of multicomponent training on PSD included only four studies, resulting in a constrained sample size and reduced reliability [30]. We also observed that, despite most studies investigating the impact of exercise on PSD [28,29,30,31], none provided comprehensive details regarding the specific exercise protocols or intervention strategies employed.

Therefore, the objective of this study is to examine the effect of exercise on depressive symptoms in stroke patients, with the aim of identifying the optimal exercise regimen for ameliorating depression in stroke patients.

## 2. Materials and Methods

### 2.1. Design

This systematic review and meta-analysis adhered rigorously to the guidelines outlined in the Preferred Reporting Items for Systematic Reviews and Meta-Analyses (PRISMA, 2020) and the Cochrane Selection Manual [32,33]. The study design was preregistered in the International Prospective Register of Systematic Reviews (PROSPERO), with the assigned identifier CRD42024595528.

### 2.2. Search Strategy

An extensive search of the literature was performed using five major electronic databases: PubMed, Web of Science, Cochrane Library, Scopus, and Embase, spanning from the inception of these databases up until 13 September 2024. The search methodology utilized a carefully designed approach, incorporating both Medical Subject Headings (MeSH) terms and relevant free-text keywords pertinent to the three core areas: stroke, exercise, and depression. Furthermore, a manual examination of reference sections from review articles was carried out to ensure the inclusion of all potentially relevant studies. Two investigators (Y.Z. and G.L.) independently conducted the search and study screening. In the event of disagreement, a third author (L.Y.) was consulted to achieve consensus.

### 2.3. Eligibility Criteria

Studies were included based on the following criteria: (1) a randomized controlled trial (RCT) design; (2) the presence of both an exercise intervention group and a control group; (3) participants had been diagnosed with stroke; and (4) depression was assessed as the primary outcome.

Studies were excluded if they met any of the following conditions: (1) published in languages other than English; (2) review articles and conference abstracts; (3) animal-based research; (4) high risk of bias or lack of access to full-text articles; (5) outcomes that could not be quantified in terms of mean and standard deviation (SD) values.

### 2.4. Data Extraction

Two investigators (Y.Z. and G.L.) independently extracted the data, with disagreements resolved through consultation with a third author (L.Y.). The extracted data comprised the following: (1) basic study information, including the first author, year of publication, country of origin, and sample size; (2) intervention specifics, such as type, duration, frequency, session duration, weekly time, intensity, and personnel involved; (3) participant characteristics, including age, body mass index (BMI), and time from diagnosis; and (4) efficacy outcomes, primarily focusing on the change in depression scores from baseline to post-intervention.

### 2.5. Methodological Quality Assessment

Two investigators (Y.Z. and G.L.) independently evaluated the risk of bias in the included studies. Discrepancies were resolved through discussion with a third reviewer (L.Y.). The Cochrane Risk of Bias tool for RCTs (RoB-2) was utilized, assessing six domains: random sequence generation, allocation concealment, blinding, missing outcome data, selective reporting of outcomes, and other potential biases [34]. Each domain was classified as “low”, “high”, or “unclear” risk [35].

### 2.6. Statistical Analysis

Given the diversity of questionnaires used to assess depression, the SMD was calculated, accompanied by their respective 95% confidence intervals (CI). In cases where studies reported standard errors (SE) or 95% CIs, the corresponding SDs were derived using established conversion methods [36]. Image data were processed using the WebPlotDigitizer (https://apps.automeris.io/wpd4/) (accessed on 9 February 2025) [36]. Heterogeneity was assessed using the *I*^2^ statistic, and when the *I*^2^ value exceeded 50%, subgroup and sensitivity analyses were conducted to explain the results.

When developing an exercise prescription, factors such as the intervention type, duration, frequency, session duration, and weekly time are typically taken into consideration. Therefore, to identify the optimal exercise regimen for alleviating depression in stroke patients, the subgroup analysis evaluating the effects of exercise on depression was based on various intervention characteristics, including type (aerobic exercise, resistance exercise, and multicomponent training), duration (<12 weeks and ≥12 weeks), frequency (<3 sessions per week and ≥3 sessions per week), session duration (<60 min and ≥60 min), and weekly time (<180 min and ≥180 min). Forest plots were generated using the RevMan 5.4 software, while Egger’s test, sensitivity analyses, and funnel plots were executed using Stata 18.0. Statistical significance was established at a threshold of *p* < 0.05.

## 3. Results

### 3.1. Studies Selection

**Figure 1:**
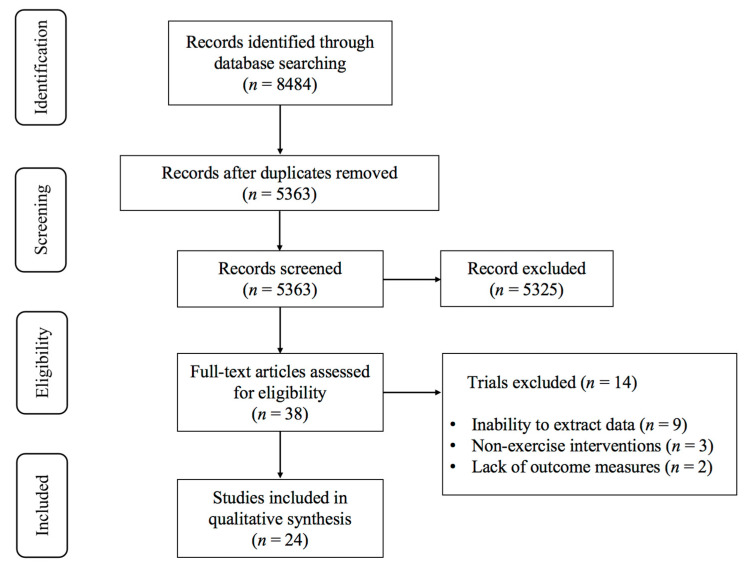
PRISMA flowchart of study selection.

During the initial database search, we identified a total of 8484 articles, as depicted in Figure 1. Following deduplication, 5363 articles remained for further consideration. Title and abstract screening excluded 5325 studies that did not meet the inclusion criteria. For the remaining 38 articles, a full-text screening was conducted, resulting in the exclusion of 14 articles due to the following reasons: (1) inability to extract data (*n* = 9); (2) lack of outcome measures (*n* = 2); and (3) non-exercise interventions (*n* = 3). Ultimately, 24 studies [23,24,25,26,27,37,38,39,40,41,42,43,44,45,46,47,48,49,50,51,52,53,54,55] were incorporated into the systematic review and meta-analysis.

### 3.2. Characteristics of the Included Studies

The primary characteristics of the included studies are summarized in Table S1. These 24 studies encompassed 28 trials spanning a publication period from 2006 [41] to 2024 [44] and were conducted across diverse countries, such as Brazil [37,38,39,40], the United States [41,42,43], China [44,45,46], the Netherlands [26,47,48], Norway [27,49], Canada [24,50], Australia [51,52], New Zealand [53], Sweden [54], South Korea [25], Croatia [23], and Burundi [55]. Sample sizes ranged from 22 to 362 participants, with the time from stroke onset varying widely among the patients, from less than 7 days to over 10 years. The studies included 1369 males and 898 females, with ages spanning from 48 to 77.7 years. Among the patients, 1102 had ischemic strokes, 281 had hemorrhagic strokes, 1 had a combination of both ischemic and hemorrhagic strokes, and the stroke type was unspecified for 22 participants. Eight studies [23,27,42,50,51,52,53,54] did not report specific details of the type of stroke in the subjects. In terms of stroke laterality, 570 participants had strokes in the left hemisphere, 567 had strokes in the right hemisphere, and 147 had strokes in other brain regions. Additionally, 11 studies [24,27,37,39,40,42,44,50,52,53,54] did not report specific details about the stroke laterality in their participants.

Regarding outcome measures, eight studies [23,24,27,47,48,49,53,55] utilized the Hospital Anxiety and Depression Scale (HADS) scale, four studies [25,42,43,52] employed the Center for Epidemiologic Studies Depression Scale (CES-D) scale, four studies [37,39,40,46] used the Beck Depression Inventory (BDI) scale, four studies [41,50,51,54] applied the Geriatric Depression Scale (GDS) scale, two studies [44,45] utilized the Hamilton Depression Scale (HAMD) scale, and one study each used the Patient Health Questionnaire-9 (PHQ-9) [38] and Patient-Reported Outcomes Measurement Information System (PROMIS) [48] scales.

In terms of intervention types, 10 studies [24,25,37,38,40,42,43,44,45,51] incorporated aerobic exercise; 2 studies [39,52] implemented resistance exercise; 10 studies [23,26,43,46,47,48,50,53,54,55] employed multicomponent training, which encompassed combinations of the following: aerobic and resistance exercises [53], physical activity and functional performance training [54], resistance exercise and balance training [41], resistance exercise paired with balance and core training [23,50], cycling and coordination training [26,55], graded task-oriented circuit training and group games [47], Tai Chi integrated with balance training [46], aerobic exercise and range of movement training [43], and physical exercise and mobility training [48]; 2 studies [24,49] utilized high-intensity interval training (HIIT), and 1 study [27] did not specify the intervention type. All exercise interventions were supervised by physical therapists, occupational therapists, exercise coaches, or exercise physiologists. However, most studies lacked detailed information on the instructors’ qualifications or experience. The intervention programs varied in length, spanning from a minimum of 3 weeks to a maximum of 18 months, with session durations falling between 30 and 120 min. However, two studies [41,52] did not provide details regarding the duration of each session. The frequency of intervention per week ranged from 2 to 14 times. We also calculated the weekly time based on the frequency and session duration. The weekly time ranged from 90 to 420 min.

### 3.3. Main Effects

**Figure 2:**
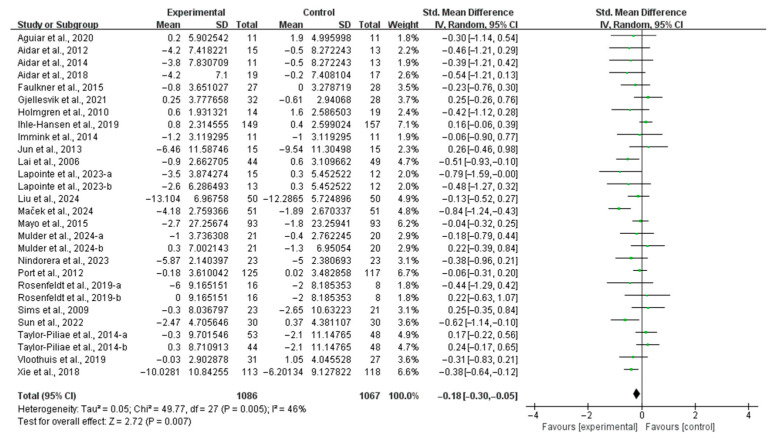
Results of the meta-analysis of the effect of exercise on depression in post-stroke patients [23,24,25,26,27,37,38,39,40,41,42,43,44,45,46,47,48,49,50,51,52,53,54,55].

When compared with the control group, exercise significantly alleviated depressive symptoms in stroke patients (SMD, −0.18; 95% CI, −0.30 to −0.05; *p* = 0.007; *I*^2^ = 46%; Figure 2).

### 3.4. Subgroup Analysis

**Figure 3:**
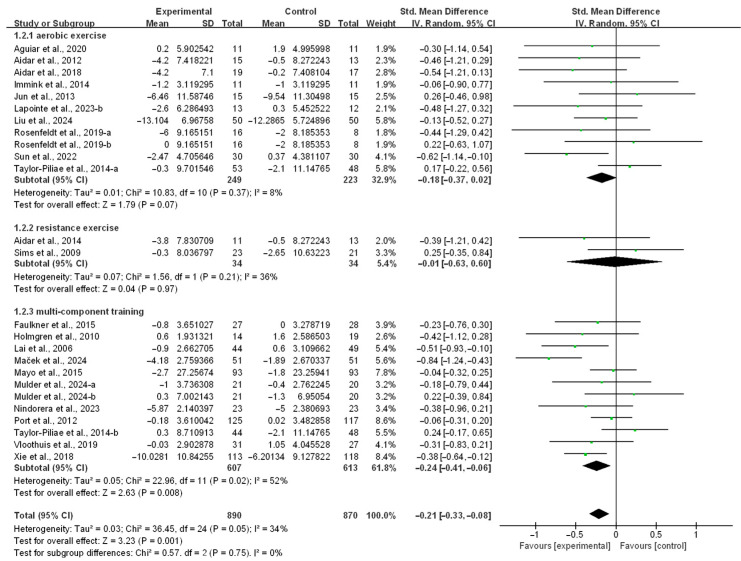
Results of the meta-analysis on the effects of different intervention approaches on depression in post-stroke patients [23,24,25,26,37,38,39,40,41,42,43,44,45,46,47,48,50,51,52,53,54,55].

The analysis was stratified by intervention type, revealing that only multicomponent training significantly alleviated depressive symptoms in stroke patients (SMD, −0.24; 95% CI, −0.41 to −0.06; *p* = 0.008; *I*^2^ = 52%; Figure 3). Aerobic exercise (SMD, −0.18; 95% CI, −0.37 to 0.02; *p* = 0.07; *I*^2^ = 8%; Figure 3) and resistance exercise (SMD, −0.01; 95% CI, −0.63 to 0.60; *p* = 0.97; *I*^2^ = 36%; Figure 3) did not demonstrate significant effects in alleviating depressive symptoms in stroke patients.

**Figure 4:**
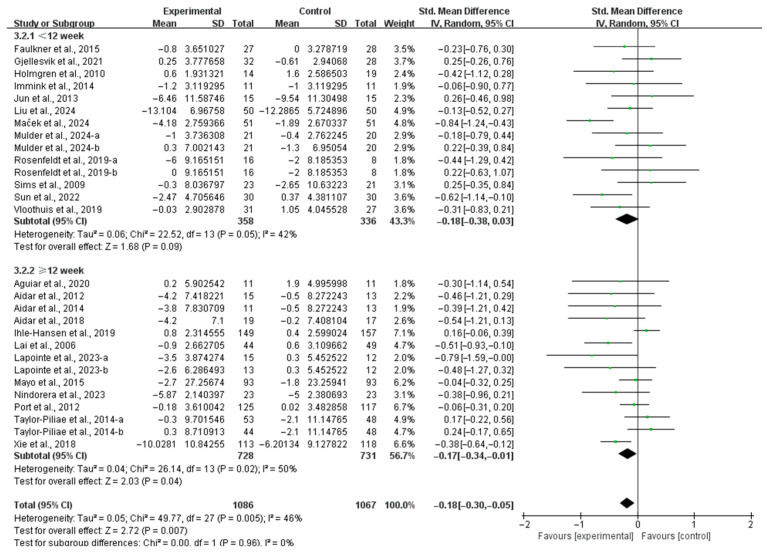
Results of the meta-analysis on the effects of intervention duration on depression in post-stroke patients [23,24,25,26,27,37,38,39,40,41,42,43,44,45,46,47,48,49,50,51,52,53,54,55].

Additionally, when stratified by intervention duration, interventions lasting ≥12 weeks significantly alleviated depressive symptoms (SMD, −0.17; 95% CI, −0.34 to −0.01; *p* = 0.04; *I*^2^ = 50%; Figure 4), while interventions lasting <12 weeks did not significantly alleviate depressive symptoms in stroke patients (SMD, −0.18; 95% CI, −0.38 to 0.03; *p* = 0.09; *I*^2^ = 42%; Figure 4).

**Figure 5:**
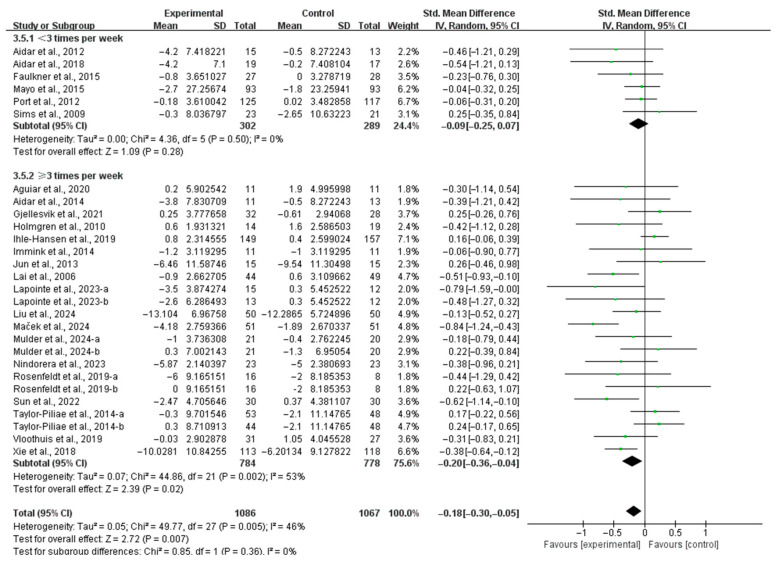
Results of the meta-analysis on the effects of intervention frequency on depression in post-stroke patients [23,24,25,26,27,37,38,39,40,41,42,43,44,45,46,47,48,49,50,51,52,53,54,55].

In subgroup analysis based on frequency, interventions conducted ≥3 times per week significantly alleviated depressive symptoms (SMD, −0.20; 95% CI, −0.36 to −0.04; *p* = 0.02; *I*^2^ = 53%; Figure 5), while interventions conducted <3 times per week did not significantly impact depressive symptoms in stroke patients (SMD, −0.09; 95% CI, −0.25 to 0.07; *p* = 0.28; *I*^2^ = 0%; Figure 5).

**Figure 6:**
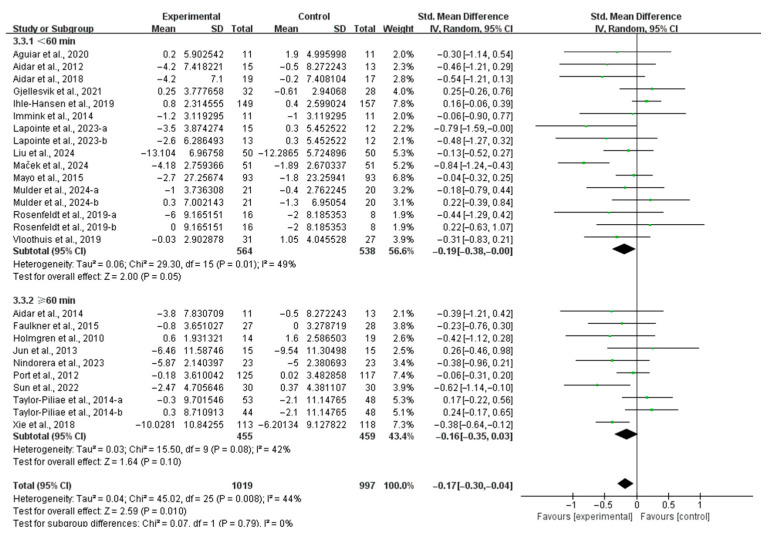
Results of the meta-analysis on the effects of different intervention durations on depression in post-stroke patients [23,24,25,26,27,37,38,39,40,42,43,44,45,46,47,48,49,50,51,53,54,55].

Considering the session duration, interventions conducted for <60 min per session significantly alleviated depressive symptoms (SMD, −0.19; 95% CI, −0.38 to −0.00; *p* = 0.05; *I*^2^ = 49%; Figure 6), while interventions conducted for ≥60 min per session did not significantly alleviate depressive symptoms in stroke patients (SMD, −0.16; 95% CI, −0.35 to 0.03; *p* = 0.10; *I*^2^ = 42%; Figure 6).

**Figure 7:**
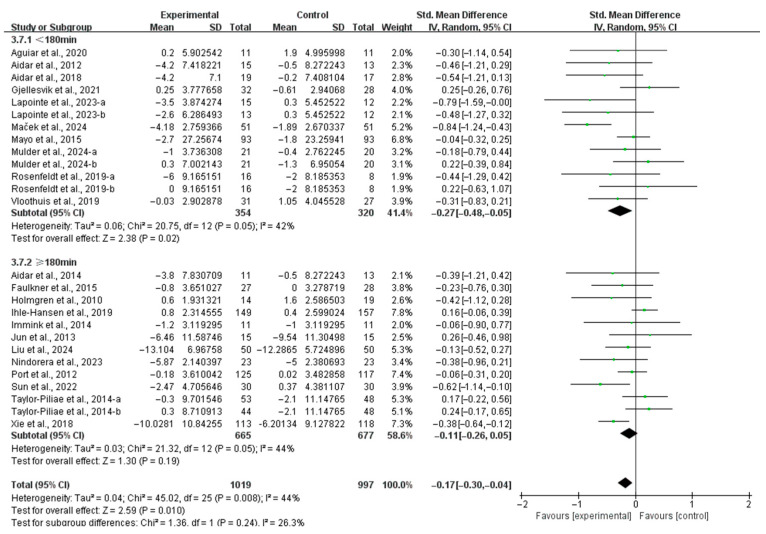
Results of the meta-analysis on the effects of the duration of weekly interventions on depression in post-stroke patients [23,24,25,26,27,37,38,39,40,42,43,44,45,46,47,48,49,50,51,53,54,55].

Finally, when stratified by weekly time, interventions conducted for <180 min per week significantly alleviated depressive symptoms (SMD, −0.27; 95% CI, −0.48 to −0.05; *p* = 0.02; *I*^2^ = 42%; Figure 7). Conversely, interventions conducted for ≥180 min per week did not show a significant improvement in depressive symptoms in stroke patients (SMD, −0.11; 95% CI, −0.26 to 0.05; *p* = 0.19; *I*^2^ = 44%; Figure 7).

### 3.5. Risk of Bias

The methodological rigor of the included studies was evaluated using the RoB-2 tool, which examines potential biases related to selection, performance, detection, attrition, reporting, and other sources. Based on the evaluation (Figure S1), the studies were classified into three quality tiers: low, moderate, and high risk of bias. Specifically, 4 studies [23,42,54,55] were assessed as having a low risk of bias, 19 studies [24,25,26,27,38,39,40,41,43,44,45,46,47,48,49,50,51,52,53] had a moderate risk, and 1 study [37] had a high risk of bias.

### 3.6. Publication Bias

To investigate potential publication bias, a funnel plot analysis and Egger’s test were performed on the 24 studies examining depression outcomes. Visual inspection of the funnel plot (Figure S2) and the results of Egger’s test (*t* = −1.34; *p* = 0.192; Table S2) did not indicate any significant publication bias.

### 3.7. Sensitivity Analyses

The sensitivity analysis revealed no alternation in the direction or magnitude of the overall effect of exercise on depressive symptoms in stroke patients (Figure S3), regardless of the exclusion of any of the included studies.

## 4. Discussion

### 4.1. Main Findings

The aim of this study was to examine the effect of exercise on depressive symptoms in stroke patients and to ascertain the optimal exercise regimen for this patient population. Our results revealed that exercise significantly alleviated depressive symptoms in stroke patients, with multicomponent training being the most effective intervention type. Subgroup analysis further indicated that exercise conducted for <60 min, ≥3 times per week, and <180 min per week exhibited more pronounced effects in alleviating depressive symptoms in stroke patients.

### 4.2. Effects of Exercise on Depressive Symptoms in Stroke Patients

The results demonstrated that exercise significantly alleviates depressive symptoms in stroke patients, aligning with prior research. For example, Eng et al. [28] reported that exercise interventions lasting at least four weeks can significantly alleviate PSD. Similarly, a meta-analysis by Li et al. [31], which focused on middle-aged and elderly stroke patients, revealed that exercise significantly alleviated depressive symptoms in patients with mild stroke.

Exercise has been shown to exert a beneficial effect on depressive symptoms in stroke patients. This is attributed to its multifaceted mechanisms of action on depressive symptoms. At the molecular level, aerobic exercise stimulates the metabolism of central nervous system neurotransmitters by elevating brain concentrations of serotonin (5-HT) and norepinephrine (NE) and by augmenting dopamine release via the activation of the phosphatidylinositol C signaling pathway [56]. These neurochemical alterations mirror the mechanisms of antidepressive medications [57].

With respect to neuroplasticity, combinations of aerobic and resistance exercises have been demonstrated to foster hippocampal neurogenesis by regulating the expression of brain-derived neurotrophic factor (BDNF) and vascular endothelial growth factor (VEGF) [58,59,60]. Additionally, aerobic exercise has been found to preserve the structural integrity of the hippocampus and white matter [61] and to augment the thickness of the anterior cingulate cortex (ACC). Furthermore, it enhances synaptic plasticity, including dendritic remodeling, which subsequently bolsters cognitive function and alleviates depressive symptoms [62].

Exercise’s immunomodulatory effects are manifested through its ability to upregulate the expression of peroxisome proliferator-activated receptor gamma coactivator-1 alpha (PGC-1α) [63], thereby reducing levels of pro-inflammatory cytokines such as interleukin-1 (IL-1), interleukin-6 (IL-6), and tumor necrosis factor-α (TNF-α) [64] and alleviating neuroinflammation. Furthermore, aerobic exercise has been demonstrated to elevate the levels of kynurenine aminotransferase in muscle tissue, promoting the synthesis of neuroprotective compounds and enhancing the antioxidative capacity, which mitigates oxidative stress-induced damage [65].

Regarding endocrine regulation, aerobic exercise has been shown to improve hypothalamic–pituitary–adrenal (HPA) axis function, elevate dopamine levels in the prefrontal cortex, and modulate the expression of glucocorticoid receptors, thereby enhancing stress adaptability [66].

From a broader physiological standpoint, aerobic exercise has been proven to enhance cardiovascular function and cerebral blood circulation [67], whereas resistance exercise has been shown to forestall muscle atrophy, optimize cytokine responses, and augment muscle strength and lean body mass [68,69]. These changes collectively facilitate brain function recovery. From a psychological perspective, exercise has been demonstrated to enhance self-efficacy and mental resilience, thereby improving symptoms of anxiety and fatigue [70]. Notably, group exercise, with its social interactions, can effectively alleviate feelings of isolation and helplessness, emerging as a unique strategy for alleviating depressive symptoms [71].

However, some studies have reported inconsistent findings. A study by Song et al. [30], focusing on middle-aged and elderly stroke patients, revealed no statistically significant improvement in PSD with multicomponent training. This discrepancy may stem from the control group’s high level of physical activity, which potentially obscured the intervention effect. Similarly, a non-pharmacological treatment study by Lee et al. [16] observed no significant impact of exercise on PSD, potentially due to the limited number of studies included (only six), thereby affecting statistical power. It is noteworthy that all studies reporting no significant effects exhibited a slight tendency toward positive outcomes. The present study also identified a modest impact of exercise on PSD. Given the substantial heterogeneity among studies, we conducted subgroup analyses to further elucidate this relationship.

### 4.3. Effects of Various Exercise Moderators on Depressive Symptoms in Stroke Patients

The results of the subgroup analysis revealed that multicomponent training stands out as the most potent intervention among various modalities. Specifically, in terms of intervention specifics, it has been established that engaging in exercise at least three times per week, with sessions not exceeding 60 min and a cumulative weekly time of no more than 180 min, yields the most favorable outcomes in treating depressive symptoms in stroke patients.

Regarding the types of exercise interventions, our findings reveal that multicomponent training is effective in alleviating depressive symptoms in stroke patients. Conversely, single-mode aerobic or resistance exercise failed to produce significant results, a finding that aligns with previous studies. For instance, Sliva et al. [72] demonstrated that multicomponent training outperformed chronic physical exercise in alleviating depressive symptoms in elderly individuals residing in nursing homes. Furthermore, a meta-analysis conducted by Li et al. [31] corroborated that multicomponent training had a superior effect in alleviating depressive symptoms in middle-aged and elderly stroke patients compared with single-exercise modalities.

Several factors may constrain the efficacy of single-mode exercise. Firstly, the limited number of studies on resistance exercise, coupled with inconsistent results, may undermine the statistical robustness of the findings. Secondly, mind–body exercises such as yoga and tai chi were categorized as aerobic exercise in this study, potentially obscuring their unique benefits. Notably, this finding resonates with a comprehensive analysis of quality of life in stroke patients, which indicated that aerobic exercise alone did not significantly improve quality of life [73]. Furthermore, all included studies incorporated external supervision and group-training elements, indicating that social interaction factors may introduce potential confounding variables [41].

Multicomponent training, which integrates various forms of exercise, including aerobic exercise, resistance exercise, and balance training, offers a comprehensive and adaptable training framework [30]. This approach boasts several notable advantages. Firstly, it allows for the customization of training programs tailored to each patient’s specific physical conditions and tolerance levels, with adjustable intensity based on their rehabilitation progress [74]. Secondly, compared with isolated high-intensity resistance training, multicomponent training demands less and enjoys higher patient compliance. Furthermore, its intrinsic diversity provides comprehensive physiological stimulation to the body, supporting adaptive changes within the nervous system and significantly enhancing cognitive function. In clinical settings, this integrated training method has demonstrated effectiveness in enhancing patients’ overall physical fitness while alleviating anxiety, reducing fatigue, and improving sleep quality. Moreover, it has been shown to mitigate other related symptoms, offering substantial therapeutic benefits [75,76,77].

Our study found that interventions lasting 12 weeks or more significantly alleviated depressive symptoms in stroke patients, while shorter interventions showed no significant effects. This result is supported by extensive substantial evidence from multiple studies. A systematic review indicated that 12 weeks of progressive exercise not only improves physical function and the emotional state but also maintains its effects over a 3–6 month period [78], suggesting that long-term exercise interventions may have more therapeutic effects. Furthermore, Sims et al. [52] revealed that a 6-month follow-up, requiring patients to maintain regular exercise habits, yielded more pronounced improvements in depressive symptoms than a 10-week intervention.

Regarding the mechanism underlying the effect of intervention duration, previous studies have established a multifaceted evidence base. A meta-analysis investigating the dose-response effect of exercise demonstrated that prolonged regimens exceeding 12 weeks, combined with high-intensity exercises, significantly enhanced functional mobility in stroke patients [79]. The study conducted by Li et al. [80] further supports this finding, indicating that exercise interventions lasting at least 12 weeks lead to notable improvements in cognitive function, whereas shorter interventions failed to achieve comparable outcomes. These findings indicate that inducing changes in brain structure and function may necessitate a minimum of 12 weeks of exercise, with individual patient differences and baseline physical fitness levels being essential considerations in determining the appropriate intervention duration [81,82]. Given the existing evidence, we hypothesized that long-term exercise may alleviate PSD by fostering functional adaptation and recovery.

Our results revealed that interventions occurring at least three times per week significantly alleviated depressive symptoms in stroke patients, while lower frequencies showed no remarkable effects. This finding aligns with current clinical guidelines. A systematic review of stroke patient exercise guidelines recommends engaging in at least three exercise sessions per week and incorporating multiple short, moderate-intensity exercise bouts daily (e.g., three sessions per day, each lasting 10–15 min) [83]. Furthermore, a recent meta-analysis confirmed the effectiveness of a three-times-per-week exercise regimen in enhancing overall health outcomes for stroke patients [80].

This recommendation regarding exercise frequency has been endorsed by numerous authoritative organizations. For instance, the United Kingdom Institute for Clinical Systems advises patients diagnosed with depression to engage in three to five exercise sessions per week [84]. Additionally, the American College of Sports Medicine (ACSM) recommends five sessions of low- to moderate-intensity exercise per week [85]. Specifically, clinical guidelines for stroke patients suggest initiating aerobic exercise at a frequency of three sessions per week, gradually increasing to five to seven sessions per week. Resistance exercise should begin at two sessions per week, with a subsequent increase to three sessions [86].

Benefits of increased exercise frequency stem from various mechanisms, broadly categorized as follows: (1) it fosters the development of consistent exercise habits, thereby enhancing patient compliance [35]; (2) it increases the frequency of cerebral stimulation, promoting the secretion of neurochemical substances; (3) it enhances neuroplasticity and optimizes autonomic nervous system regulation [87]. These physiological and behavioral mechanisms collectively contribute to the alleviation of PSD.

This study found that sessions lasting less than 60 min were associated with significant improvements in depressive symptoms in stroke patients. Conversely, interventions with a minimum duration of 60 min had no notable impact on depressive symptoms. This aligns with previous research showing that 40 min of aerobic exercise significantly improves patients’ walking ability, quality of life, and depressive symptoms [38]. In contrast, 100 min of personalized group training yielded no significant effects [54], indicating that extending the session duration does not necessarily lead to enhanced therapeutic outcomes.

The United Kingdom Institute for Clinical Systems recommends 30 min exercise sessions, while stroke patient exercise guidelines suggest limiting each session to 20–60 min based on individual functional differences [84]. Furthermore, the benefit of shorter exercise periods may be related to the physiological characteristics of stroke patients, who expend approximately twice the energy per step compared with healthy individuals [88]. Therefore, prolonged exercise may lead to over-fatigue and diminish the antidepressive effects.

The World Health Organization (WHO) 2020 guidelines on physical activity and sedentary behavior advise adults to engage in 150–300 min of moderate-intensity exercise or 75–150 min of vigorous-intensity exercise per week [89]. Similarly, ACSM recommends that adults engage in 150 min of exercise per week, including aerobic, resistance, and neuromotor activities [85].

The findings of this study indicated that exercise totaling less than 180 min per week is very effective in treating PSD, while weekly exercise of 180 min or more showed no significant effect. The combination of at least three sessions per week and sessions lasting less than 60 min was found to significantly alleviate depressive symptoms in stroke patients. Notably, stroke patients often have lower exercise tolerance, which can lead to fatigue and exacerbate depressive symptoms [90]. Therefore, for stroke patients, increasing the frequency of exercise rather than extending the session duration may be a more effective strategy in achieving the weekly exercise target.

### 4.4. Limitations

This study was subject to several limitations that warrant acknowledgement. Firstly, the implementation of a complete blinding design was infeasible in the RCTs assessing exercise interventions, potentially introducing subjective bias during the evaluation phase. Secondly, the studies included in this systematic review focused on depressive symptoms rather than clinically diagnosed depression, as per the Diagnostic and Statistical Manual of Mental Disorders (DSM). Additionally, the majority of the included studies lacked comprehensive documentation regarding the intensity of the exercise interventions, thereby posing challenges in assessing the precise impact of exercise intensity on the amelioration of PSD. Furthermore, participants underwent rehabilitation or pharmacological treatments alongside exercise interventions. The grouping was exclusively based on exercise interventions, without examining the potential synergistic effects between exercise and other treatment modalities. Moreover, several of the included studies reported comorbidities among participants, yet these comorbidities were not considered as potential confounding factors influencing the outcomes of exercise interventions. However, comorbidities may impact the effectiveness of exercise interventions in stroke patients, particularly in terms of the improvement of depressive symptoms. Future research should focus on the potential confounding effects of comorbidities on the outcomes of exercise interventions and further explore the combined effects of exercise and other therapeutic strategies to provide more comprehensive treatment approaches.

### 4.5. Practical Implications

This study highlights the importance of exercise in managing depressive symptoms in stroke patients. Healthcare providers should incorporate exercise into stroke rehabilitation programs, especially multicomponent training, which has demonstrated significant benefits. The recommendation is for stroke patients to exercise at least three times a week, with sessions lasting no more than 60 min each and totaling no more than 180 min per week. As the patient progresses, the frequency can be gradually increased based on their abilities and needs while still ensuring safety. Clinicians should monitor the patient’s progress and adjust the exercise plan as necessary to maintain effectiveness. Exercise provides a valuable non-pharmacological approach to improving mental health and overall well-being for stroke patients.

## 5. Conclusions

Exercise has been shown to alleviate depressive symptoms in stroke patients, with multicomponent training emerging as a potentially optimal intervention. To alleviate depressive symptoms, this meta-analysis provides evidence-based recommendations for clinicians, advising stroke patients to engage in exercise at least 3 times weekly, with individual sessions not exceeding 60 min. When increasing the frequency of exercise, the cumulative weekly time should ideally remain below 180 min for optimal outcomes.

## Data Availability

All data generated or analyzed during this study are included in the article/Supplementary Materials.

## Supplementary Materials

The following supporting information can be downloaded at: https://www.mdpi.com/article/10.3390/life15020285/s1, Figure S1: Results of the Cochrane risk of bias tool; Figure S2: Funnel plot; Figure S3: Sensitivity analyses results; Table S1: Characteristics of the studies included in this meta-analysis; Table S2: Results of Egger’s test.
