# Supplementary material for: Effects of Exercise on Post-Stroke Depression: A Systematic Review and Meta-Analysis of Randomized Controlled Trials
Source: Life (Basel). 2025 Feb 12;15(2):285. doi: 10.3390/life15020285 (PMC11857396; doi:10.3390/life15020285)

Supplemental material

Effects of exercise on post-stroke depression: a systematic review and meta-analysis of randomized controlled trials

Table S1. Characteristics of the studies included in this meta-analysis....2

Table S2. Results of Egger's test....5

Figure S1. Results of Cochrane risk of bias tool.... 6

Figure S2. Funnel plot.... 7

Figure S3. Sensitivity analysis results....8

Table S1. Characteristics of the studies included in this meta-analysis

Study	Country	Sample size	Stage of illness	Gender (male/female)	Mean age(y)	Intervention	Type of intervention	Minutes per session	Frequency (times / week)	Duration (weeks)	Weekly time	Intensity	Supervision	Depression outcomes
Aguiar et al., 2020	Brazil	Int: 11<br>Con: 11	Int: 51 ± 68<br>Con: 44 ± 26 (month)	Int: 8 / 3<br>Con: 8 / 3	Int: 52 ± 11<br>Con: 48 ± 10	Treadmill training	Aerobic	40	3	12	120	HRR 60-80%	Yes	PHQ
Aidar et al., 2012	Brazil	Int: 15<br>Con: 13	> 12 months	Int: 10 / 5<br>Con: 9 / 4	Int: 50.3 ± 9.1<br>Con: 52.5 ± 7.7	Aquatic physical activity program	Aerobic	50	2	12	100	Borg 12-17	Yes	BDI
Aidar et al., 2014	Brazil	Int: 11<br>Con: 13	> 12 months	Int: 6 / 5<br>Con: 9 / 4	Int: 51.7 ± 8.0<br>Con: 52.5 ± 7.7	Squat, Bench press, Leg press, Military press, Crunch, Lat pulldowns, Lunges	Resistance	60	3	12	180	OMNI 6-8	Yes	BDI
Aidar et al., 2018	Brazil	Int: 19<br>Con: 17	> 12 months	Int: 10 / 9<br>Con: 9 / 8	Int: 51.8 ± 8.5<br>Con: 52.7 ± 6.7	Aquatic physical activity program	Aerobic	50	2	12	100	Moderate	Yes	BDI
Faulkner et al., 2015	New Zealand	Int: 27<br>Con: 28	< 7 days	Int: 15 / 12<br>Con: 14 / 14	Int: 65 ± 11<br>Con: 68 ± 10	Aerobic exercise, Resistance training, Core stability, Posture training	Multicomponent	90	2	8	180	HR_{max} 50-85%	Yes	HADS
Gjellesvik et al., 2021	Norway	Int: 36<br>Con: 34	Int: 25.4 ± 14.5<br>Con: 27.4 ± 14.7 (month)	Int: 21 / 15<br>Con: 20 / 14	Int: 57.6 ± 9.2<br>Con: 58.7 ± 9.2	HIIT	HIIT	35	3	8	105	HR_{peak} 85–95%	Yes	HADS
Holmgren et al., 2010	Sweden	Int: 15<br>Con: 19	Int: 139.7 ± 37.3<br>Con: 126.8 ± 28.2 (day)	Int: 9 / 6<br>Con: 12 / 7	Int: 77.7 ± 7.6<br>Con: 79.2 ± 7.5	Group personalized training	Multicomponent	100	3	5	300	High	Yes	GDS
Ihle-Hansen et al., 2019	Norway	Int: 177<br>Con: 185	> 3 months	Int: 99 / 78<br>Con: 120 / 65	Int: 71.4 ± 11.3<br>Con: 72.0 ± 11.3	Personalized physical activity	NA	49	7	18month	345	Moderate-high	Yes	HADS

Immink et al., 2014	Australia	Int: 11 Con: 11	Int: 81.6 ± 77.5 Con: 23.3 ± 12.5 All: 52.5 ± 61.9 (month)	Int: 6 / 5 Con: 3 / 8	Int: 56.1 ± 13.6 Con: 63.2 ± 17.4 All: 59.6 ± 15.7	Yoga	Aerobic	47	7	10	330	Low	Yes	GDS
Jun et al., 2013	Korea	Int: 15 Con: 15	< 2 weeks	Int: 6 / 9 Con: 9 / 6	Int: 60.7 ± 8.59 Con: 55.1 ± 17.23	Music and movement therapy (MMT)	Aerobic	60	3	8	180	NA	Yes	CES-D
Lai et al., 2006	United States	Int: 44 Con: 49	Int: 77.5 ± 28.7 Con: 74.1 ± 27.2 (day)	Int: 23 / 21 Con: 27 / 22	Int: 68.5 ± 9.0 Con: 70.4 ± 11.3	Progressive structured exercise program	Multicomponent	NA	3	12	NA	NA	Yes	GDS
Lapointe et al., 2023-a	Canada	Int: 19 Con: 17	Int: 37.3 ± 61.6 Con: 29.3 ± 39.1 (month)	Int: 13 / 6 Con: 10 / 7	Int: 71.8 ± 9.9 Con: 69.6 ± 10.7	HIIT + MICT	HIIT + MICT	40	3	18	120	PPO 95%	Yes	HADS
Lapointe et al., 2023-b	Canada	Int: 16 Con: 17	Int: 51.8 ± 78.7 Con: 29.3 ± 39.1 (month)	Int: 10 / 6 Con: 10 / 7	Int: 65.6 ± 11.3 Con: 69.6 ± 10.7	MICT	Aerobic	40	3	18	120	PPO 50%	Yes	HADS
Liu et al., 2024	China	Int: 50 Con: 50	Int: 85.12 ± 44.11 Con: 77.98 ± 39.22 (day)	Int: 31 / 19 Con: 29 / 21	Int: 58.86 ± 10.83 Con: 56.22 ± 11.54	Baduanjin + Rational emotive behavior therapy (REBT)	Aerobic	30	14	8	420	NA	Yes	HAMD-17
Maček et al., 2024	Croatia	Int: 51 Con: 51	7 - 45 days	Int: 14 / 37 Con: 22 / 29	Int: 68.2 ± 7.26 Con: 68.7 ± 6.80	Standard neurophysiotherapy + Group exercise	Multicomponent	45	3	3	135	NA	Yes	HADS
Mayo et al., 2015	Canada	Int: 93 Con: 93	Int: 2.5 ± 2.2 Con: 3.1 ± 3.1 (year)	Int: 57 / 36 Con: 56 / 37	Int: 61 ± 12 Con: 65 ± 11	Aerobic, Core training, Balance, Flexibility	Multicomponent	45	2	12	90	NA	Yes	S-GDS
Mulder et al., 2024-a	Netherlands	Int: 21 Con: 20	Int: 55.2 ± 16.6 Con: 56.8 ± 17.1 (day)	Int: 11 / 10 Con: 12 / 8	Int: 61.3 ± 11.5 Con: 63.0 ± 8.9	Armed4Stroke	Multicomponent	30	5	8	150	NA	Yes	HADS
Mulder et al., 2024-b	Netherlands	Int: 21 Con: 20	Int: 55.2 ± 16.6 Con: 56.8 ± 17.1 (day)	Int: 11 / 10 Con: 12 / 8	Int: 61.3 ± 11.5 Con: 63.0 ± 8.9	Armed4Stroke	Multicomponent	30	5	8	150	NA	Yes	PROMIS
Nindorera et al., 2023	Burundi	Int: 23 Con: 23	Int: 24 Con: 21 (month)	Int: 18 / 5 Con: 18 / 5	Int: 50.9 ± 10.7 Con: 50.1 ± 11.2	Multimode circuit training plan	Multicomponent	120	3	12	360	HR_{max} 50-75%	Yes	HADS

Port et al., 2012	Netherlands	Int: 126<br>Con: 124	Int: 91 ± 42<br>Con: 103 ± 51<br>(day)	Int: 82 / 44<br>Con: 80 / 44	Int: 56 ± 10<br>Con: 58 ± 10	Task-Oriented Circuit Training	Multicomponent	90	2	12	180	NA	Yes	HADS
Rosenfeldt et al., 2019-a	United States	Int: 16<br>Con: 8	Int: 12<br>Con: 17<br>(month)	Int: 12 / 4<br>Con: 7 / 1	Int: 51 ± 12<br>Con: 58 ± 12	Cycle	Aerobic	45	3	8	135	HRR<br>60-80%	Yes	CES-D
Rosenfeldt et al., 2019-b	United States	Int: 16<br>Con: 8	Int: 16<br>Con: 17<br>(month)	Int: 10 / 6<br>Con: 7 / 1	Int: 60 ± 14<br>Con: 58 ± 12	Cycle	Aerobic	45	3	8	135	HRR<br>60-80%	Yes	CES-D
Sims et al., 2009	Australia	Int: 23<br>Con: 22	< 6 months	Int: 14 / 9<br>Con: 13 / 9	Int: 67.95 ± 14.76<br>Con: 66.27 ± 16.01	PRT program	Resistance	NA	2	10	NA	80%1RM	Yes	CES-D
Sun et al., 2022	China	Int: 30<br>Con: 30	Int: 7.53 ± 4.09<br>Con: 7.97 ± 5.27<br>(month)	Int: 17 / 13<br>Con: 17 / 13	Int: 62.03 ± 7.37<br>Con: 65.23 ± 6.29	Yijinjing qigong	Aerobic	60	7	3	420	low-moderate	Yes	HAMD-24
Taylor-Piliae et al., 2014-a	United States	Int: 53<br>Con: 48	Int: 39 ± 50.2<br>Con: 38.7 ± 46.7<br>(month)	Int: 34 / 19<br>Con: 23 / 25	Int: 71.5 ± 10.3<br>Con: 68.2 ± 10.3	Tai Chi	Aerobic	60	3	12	180	NA	Yes	CES-D
Taylor-Piliae et al., 2014-b	United States	Int: 44<br>Con: 48	Int: 33 ± 58.7<br>Con: 38.7 ± 46.7<br>(month)	Int: 20 / 24<br>Con: 23 / 25	Int: 69.6 ± 9.4<br>Con: 68.2 ± 10.3	Silverneakers	Multicomponent	60	3	12	180	NA	Yes	CES-D
Vloothuis et al., 2019	Netherlands	Int: 32<br>Con: 34	Int: 37<br>Con: 36<br>(day)	Int: 21 / 11<br>Con: 20 / 14	Int: 60.53 ± 14.82<br>Con: 59.26 ± 15.01	CARE4STROKE program	Multicomponent	30	5	8	150	NA	Yes	HADS
Xie et al., 2018	China	Int: 120<br>Con: 124	Int: 14.5 ± 18.1<br>Con: 14.3 ± 22.1<br>(month)	Int: 83 / 37<br>Con: 99 / 25	Int: 60.9 ± 8.7<br>Con: 60.1 ± 8.6	Tai Chi	Multicomponent	60	5	12	300	NA	Yes	BDI

Abbreviation: Int, intervention groups; Con, control groups; BMI, body mass index; HIIT, high-intensity interval training; MICT, moderate-intensity continuous training; NA, not available; HRR, heart rate reserve; Borg, the Borg RPE Scale; OMNI, the OMNI Perceived Exertion Scale; HR_{max}, maximum heart rate; HR_{peak}, peak heart rate; PPO, peak power output; 1RM, 1 repetition maximum; Moderate-high, moderate to high intensity exercise; low-moderate, low to moderate intensity exercise; Yes, intervention process is monitored; BDI, the Beck Depression Inventory; PHD, the Patient Health Questionnaire; HADS, the Hospital Anxiety and Depression Scale; GDS, the Geriatric Depression Scale; S-GSD, the Stroke-Specific Geriatric Depression Scale; CES-D, the Epidemiological Studies Depression Scale; HAMD-17, the Hamilton Depression Scale-17; HAMD-24, the Hamilton Depression Scale-24; PROMIS, the Patient-Reported Outcomes Measurement Information System

Table S2. Results of Egger's test

Std_EFF	Coef.	Std. Err.	t	p > |t|	95% CI
Slope	0.0331296	0.1426561	0.23	0.818	-0.2601043, -0.3263635
Bias	-0.8307303	0.6194759	-1.34	0.192	-2.104081, 0.4426207

Abbreviations: Coef, coefficient; Std. Err, standard error; t, *t*-test statistic; p, probability; CI, confidence interval.

Figure S1. Results of Cochrane risk of bias tool

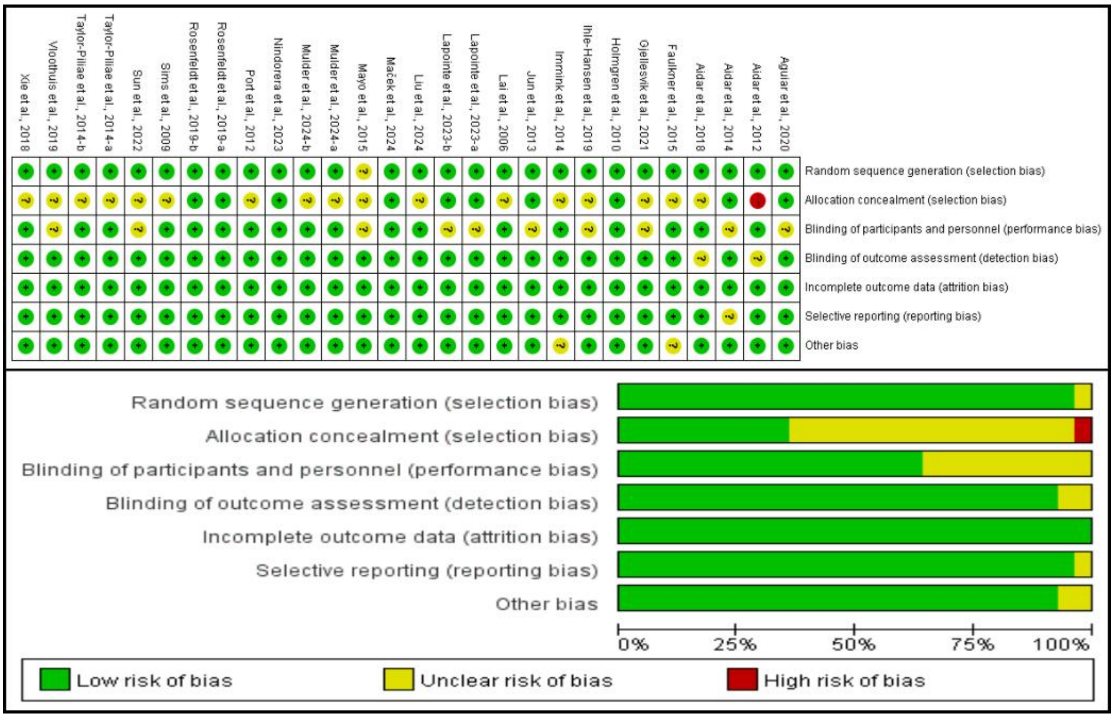

Figure S2. Funnel plot

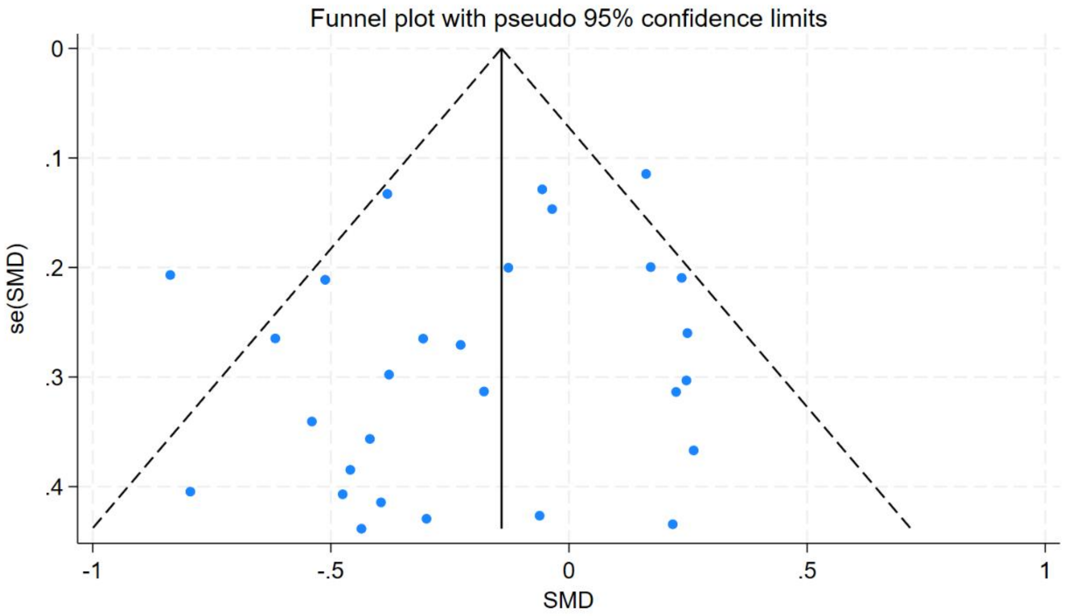

Figure S3. Sensitivity analysis results

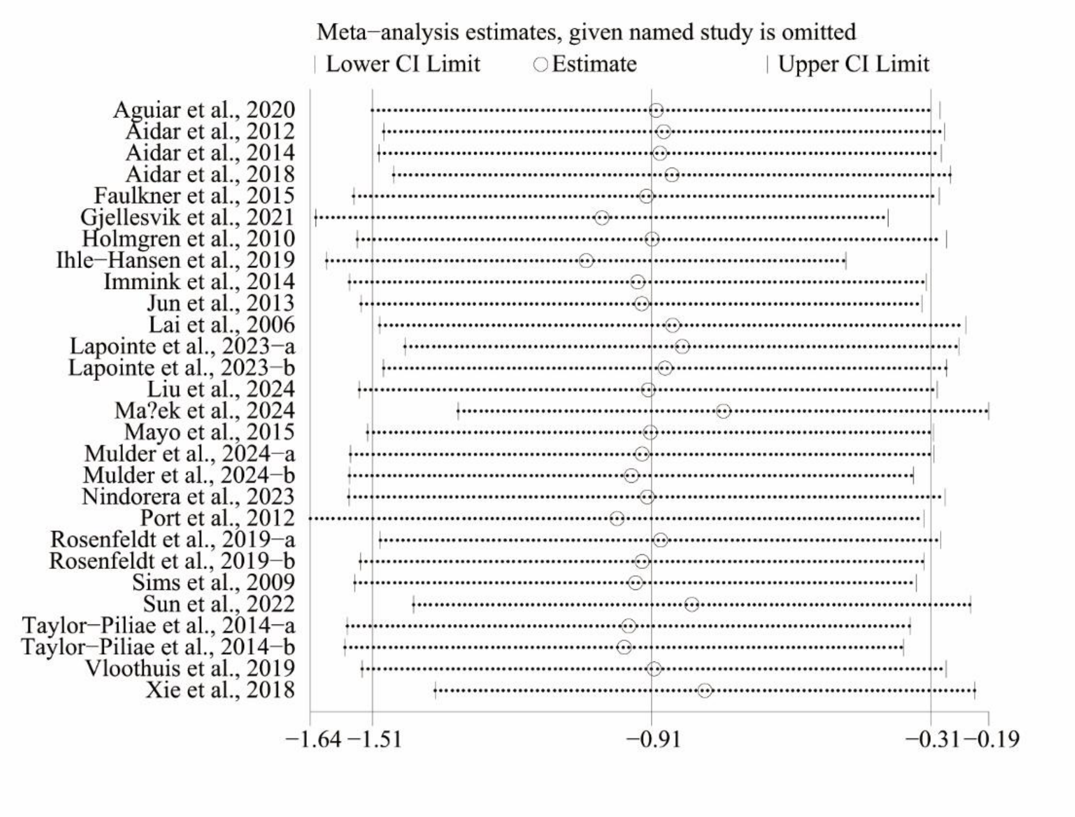

Supplement: Supplementary file 1 [file life-15-00285-s001.zip › life-3424457-supplementary.pdf]
